# Correlation between functional ability, toe flexor strength, and plantar pressure of hallux valgus in young female adults: a cross-sectional study

**DOI:** 10.1186/s13047-020-00411-1

**Published:** 2020-07-13

**Authors:** Mieko Yokozuka, Kanako Okazaki, Yuko Sakamoto, Koko Takahashi

**Affiliations:** 1 Preparing Section for New Faculty of Medical Science, Fukushima Medical University, 1 Hikariga-oka, Fukushima, 960-1295 Japan; 2 School of Nursing, Fukushima Medical University, 1 Hikariga-oka, Fukushima, 960-1295 Japan

**Keywords:** Hallux valgus, Toe flexor strength, Functional reach, Maximum step length, Plantar pressure

## Abstract

**Background:**

The prevalence of hallux valgus (HV) increases with age in females. Several studies have investigated the relationship between foot problems, including HV, and falls in older individuals. This study aimed to examine whether HV causes a decline in functional activity in young females and also evaluate the relationship between HV angle, functional activity, toe flexor strength, and plantar pressure.

**Methods:**

We assessed 94 females (mean age, 19.6 ± 1.3 years; mean body mass index, 21.2 ± 2.0 kg/m^2^) not currently receiving treatment for lower limb disease. HV angle was determined using their footprint. Functional reach (FR) and maximum step length (MSL), toe flexor strength, and plantar pressure were measured. Plantar pressure was measured during walking. We also calculated FR and the pressure in eight regions (first toe, second through fifth toes, first metatarsal, second through fourth metatarsals, fifth metatarsal, midfoot, medial heel, and lateral heel).

**Results:**

There were 39 and 55 participants in the HV and no HV groups, respectively. FR and MSL did not differ significantly between the HV and no HV groups. Toe flexor strength was significantly different between the HV and no HV groups (26.69 ± 9.68 vs. 32.19 ± 8.55, respectively) (*p* = 0.002, β = 0.206). During walking, plantar pressure was significantly lower in the second through fifth toes in the HV group (*p* = 0.005, β = 0.187). During FR, plantar pressure was significantly greater in the first metatarsal in the HV group (*p* = 0.016, β = 0.338). HV angle was negatively correlated with toe flexor strength (*r* = − 0.315, *p* = 0.002, β = 0.121) and plantar pressure during walking in the second through fifth toes (*r* = − 0.362, *p* < 0.001, β = 0.047), and positively correlated with plantar pressure during FR in the first metatarsal (*r* = 0.308, *p* = 0.002, β = 0.137). Toe flexor strength was negatively correlated with plantar pressure during FR in the second through fourth metatarsals (*r* = − 0.318, *p* = 0.002, β = 0.115), and there was a positive correlation with MSL (*r* = 0.330, *p* = 0.001, β = 0.092).

**Conclusions:**

This study confirmed that HV reduces toe flexor strength and affects forefoot pressure during walking and FR in young females. Moreover, the toe flexor strength affects MSL. Efforts to prevent the onset and deterioration of HV from a young age might help reduce the risk of falling when older.

## Background

Foot pain and deformity are associated with falls in community-dwelling older persons [1, 2]. One meta-analysis indicated that persons who have falls are more likely to have foot pain and hallux valgus (HV) with odds ratios of 1.95 and 1.89, respectively [2]. Another meta-analysis reported that the prevalence of HV in persons over 65 years was 35.7%, which was higher in females than in males [3]. Similarly, a study of 268 Japanese females aged ≥65 years living in the community, 99 (36.9%) were diagnosed with HV [4].

HV and lesser toe deformities significantly reduce the flexor strength of the hallux and lesser toes [5–7]. The relationship between HV and physical function [8], spatiotemporal gait parameters [9], balance [10, 11], timed up and go test [12], and plantar pressure have been reported [7, 8, 13–15]. However, the participants of these studies were middle-aged or older people, and the studies also included persons with mobility and ADL limitations [10, 11], with aids [5] or assistive devices [13], with foot pain or previous treatment [7], with knee osteoarthritis [8], and those with indications for surgery [14]. Therefore, factors other than HV may have been involved, and the deterioration in physical function, balance, and plantar pressure may have been due to aging.

On the other hand, about 30% of female university students in Japan are reported to have HV [16, 17]. It has been reported that young women need to be more aware of the characteristics and severity of HV [16]. To reduce the risk of falls in old age, it is important to prevent the onset and deterioration of HV among young females. To this end, it is necessary to clarify the effects of HV on motor function, even in young females. Toe flexor muscles become more active in the mid stance to toe off during walking [18, 19], and toe flexor strength was significantly correlated with the anterior limit of the functional base of support [20]. Therefore, even in young people, if the toe flexor strength is reduced due to hallux valgus, there is concern that it may affect the forward movement of the center of gravity and the motion with propulsive force in daily life.

This study aimed to compare dynamic balance in forward reaching/stepping tasks in young females with and without HV. In addition, the relationship among functional activity, toe flexor strength, and plantar pressure was assessed.

## Methods

### Participants

This cross-sectional study of 94 female university students was conducted from December 2019 to January 2020. The purpose of the study was explained to all participants in both written and verbal forms, and written informed consent was obtained. The study was approved by the University’s Ethics Committee (Approval number: 2019–187). The exclusion criteria were age > 25 years and the presence of lower limb disorders.

### Measurements

All measurements were performed with the participants barefoot.

### HV angle

Measurement of HV was conducted using a foot printer (Bauerfeind, Germany), and a footprint was taken with the participant in the standing position. The angle between the line connecting the first metatarsal head and the first proximal phalanx head, and that connecting the first metatarsal head and the calcaneus in the posterior medial malleolus position on the outline from the footprint was measured [21]. This angle is highly correlated with the HV angle on X-ray (*r* = 0.942), and a 16° HV angle on the footprint is equivalent to a 20° diagnosis of HV on X-ray [21].

### Functional reach (FR)

Participants stood upright and raised both arms to shoulder height, and the position of the top of the middle finger was marked. After that, participants stretched their arms forward as far as possible for 5 s, with their legs fixed. The maximum distance of the position of the top of the middle finger from the first position was recorded [22]. This action was performed twice, and the maximum value was taken as the result. FR was normalized by height (FR / height * 100). Studies have shown acceptable reliability of the FR [22].

### Maximum step length (MSL)

Participants were instructed to stand with both feet in the indicated position and step forward as far as possible with their right/left leg and bring the other leg up to the first leg in one step. In this position, the stepped distance was recorded. When the participant lost balance, the distance was measured again. This action was performed twice on each side and the maximum value on each side was taken as their result [23]. MSL was normalized by height (MSL / height * 100). MSL shows the best intraclass correlation coefficients [23, 24].

### Toe flexor strength

Toe flexor strength was measured using a toe grip dynamometer (T.K.K. 3364b; Takei Scientific Instruments, Niigata, Japan). The hip, knee, and ankle joints were set to 90° in a chair sitting position, and the ankle was secured with a belt to prevent the bar being pulled by the ankle dorsiflexion. The bar for measuring toe flexor strength was gripped for 3 s with maximal effort using the toes for the left and right side, respectively. Care was taken to prevent the bar being pulled by flexion of the knee joint. Toe flexor strength was measured twice on each side with a rest of 3 to 5 s to minimize the effect on the second measurement. The maximum toe flexor strength on each side was normalized by body weight (toe flexor strength / body weight * 100). The intra- and inter-rater reliability of toe grip dynamometer measurements have been validated [25].

### Plantar pressure

Plantar pressure was measured during walking and FR. The plantar pressure was measured using a foot analyzer (Gaitview UGA-526; aison, Inc., Saitama, Japan), which includes a 410 × 410 × 3-mm active area, comprising 2304 (48 × 48) force sensitive resistor sensors. The average plantar pressure was automatically applied to the foot in eight regions (first toe, second through fifth toes, first metatarsal, second through fourth metatarsals, fifth metatarsal, midfoot, medial heel, and lateral heel) using software. Plantar pressure during walking was measured using a two-step method of collecting plantar pressure values similar to plantar pressure data obtained using the midgait [26]. Walking started at approximately 80 cm from the front edge of the pressure platform and the plantar pressure of the second step was measured. Participants repeated the walking trial five times, and the average plantar pressure of five trials for each foot was calculated.

### Statistical analysis

For variables measured bilaterally (HV angle, MSL, toe flexor strength, and plantar pressure during walking and FR), data from only one limb was analyzed to maintain independence [27]. Participants were classified into HV and no HV groups. For HV participants, the foot with a greater HV angle was chosen (31 right feet and eight left feet), while for no HV participants, the right foot or left foot was chosen (27 right feet and 28 left feet) randomly using random number sampling. Tests for normality were performed with the Shapiro-Wilk test. FR, MSL, toe flexor strength, and plantar pressure were compared using the unpaired T-test or Mann-Whitney U test. Furthermore, the correlation between HV angle, FR, MSL, toe flexor strength, and plantar pressure was determined using the Spearman’s correlation coefficient, because it includes variables with a non-normal distribution. Statistical analyses were performed using SPSS for Windows (version 25.0; IBM, Armonk, NY, USA), and the significance level was set at < 5%.

## Results

The mean ± standard deviation (SD) age of the total sample was 19.6 ± 1.3 years, and the mean body mass index (BMI) was 21.2 ± 2.0 kg/m^2^. Table 1 shows the characteristics of participants. The HV group had 39 participants, whereas the no HV group had 55 participants.

**Table 1:** Characteristics of participants

	HV group (*n* = 39)	no HV group (*n* = 55)	*p*-value
HV angle (°) (range)	20.3 ± 5.1 (4–15)	11.0 ± 2.6 (16–43)	< 0.001
Age (years)	19.4 ± 1.2	19.8 ± 1.3	0.186
Height (cm)	157.4 ± 4.7	156.8 ± 5.7	0.628
Weight (kg)	52.8 ± 4.3	52.1 ± 6.2	0.521
BMI (kg/m^2^)	21.3 ± 1.8	21.2 ± 2.2	0.425

*BMI* body mass index, *HV* hallux valgus

Table 2 shows FR, MSL, toe flexor strength, and plantar pressure. FR and MSL were not significantly different in the HV and no HV groups. Toe flexor strength was significantly different at 26.69 ± 9.68 and 32.19 ± 8.55 for the HV and no HV groups, respectively (*p* = 0.002, β = 0.206). During walking, the plantar pressure in the HV group was significantly reduced for the second through fifth toes (*p* = 0.005, β = 0.187) and the second through fourth metatarsals (*p* = 0.014, β = 0.600), and was significantly increased in the lateral heel (*p* = 0.044, β = 0.603). During FR, the plantar pressure was significantly increased in the first metatarsal in the HV group (*p* = 0.016, β = 0.338).

**Table 2:** FR, MSL, toe flexor strength, and plantar pressure

	HV group (*n* = 39)	no HV group (*n* = 55)	*p*-value	β	effect size d
FR (cm/cm*100)	21.39 ± 3.61	22.15 ± 3.54	0.309	0.827	0.214
MSL (cm/cm*100)	72.10 ± 5.78	73.95 ± 6.54	0.159	0.705	0.300
Toe flexor strength (kg/bw*100)	26.69 ± 9.68	32.19 ± 8.55	0.002**	0.206	0.602
Plantar pressure (kPa)					
Gait					
T1	89.07 ± 22.65	79.41 ± 28.35	0.070	0.572	0.376
T25	61.33 ± 32.50	81.38 ± 32.50	0.005**	0.187	0.617
M1	104.74 ± 26.82	122.31 ± 52.56	0.055	0.506	0.421
M24	159.60 ± 11.88	172.92 ± 49.54	0.014*	0.600	0.370
M5	115.29 ± 24.03	121.20 ± 20.66	0.205	0.762	0.264
MF	102.91 ± 29.44	96.37 ± 39.01	0.379	0.854	0.189
MH	162.05 ± 7.96	163.01 ± 8.35	0.576	0.914	0.118
LH	168.63 ± 11.93	164.29 ± 11.69	0.044*	0.603	0.368
FR					
T1	59.23 ± 54.25	56.15 ± 49.83	0.985	0.941	0.059
T25	53.30 ± 39.26	62.64 ± 46.33	0.343	0.829	0.218
M1	105.82 ± 42.92	85.09 ± 37.44	0.016*	0.338	0.515
M24	133.13 ± 34.33	128.12 ± 29.50	0.220	0.888	0.157
M5	97.78 ± 40.45	105.52 ± 31.77	0.323	0.829	0.213
MF	70.32 ± 26.10	58.23 ± 31.04	0.050	0.487	0.422
MH	103.77 ± 34.39	110.34 ± 32.78	0.351	0.848	0.195
LH	103.70 ± 34.35	112.59 ± 30.71	0.347	0.757	0.273

*FR* functional reach, *MSL* maximum step length, *HV* hallux valgus, *T1* first toe, *T25* second through fifth toes, *M1* first metatarsal, *M24* second through fourth metatarsals, *M5* fifth metatarsal, *MF* midfoot, *MH* medial heel, *LH* lateral heel

* *p* < 0.05, ** *p* < 0.01

Table 3 shows the relationship between HV angle and toe flexor strength. HV angle was negatively correlated with toe flexor strength (*r* = − 0.315, *p* = 0.002, β = 0.121), plantar pressure during walking in the second through fifth toes (*r* = − 0.362, *p* < 0.001, β = 0.047) and the second through fourth metatarsals (*r* = − 0.242, *p* = 0.019, β = 0.342), and there was a positive correlation with plantar pressure during walking in the first toe (*r* = 0.253, *p* = 0.014, β = 0.301) and the lateral heel (*r* = 0.301, *p* = 0.003, β = 0.154) and during FR in the first metatarsal (*r* = 0.308, *p* = 0.002, β = 0.137) and midfoot (*r* = 0.212, *p* = 0.040, β = 0.459). Toe flexor strength was negatively correlated with plantar pressure during walking in midfoot (*r* = − 0.211, *p* = 0.041, β = 0.463) and during FR in the second through fourth metatarsals (*r* = − 0.318, *p* = 0.002, β = 0.115) and midfoot (*r* = − 0.348, *p* = 0.001, β = 0.064), and there was a positive correlation with MSL (*r* = 0.330, *p* = 0.001, β = 0.092), during walking in the second through fifth toes (*r* = 0.226, *p* = 0.029, β = 0.403), and during FR the first toe (*r* = 0.223, *p* = 0.031, β = 0.415).

**Table 3:** Correlations between HV angle and toe flexor strength and FR, MSL and plantar pressure

	HV angle			Toe flexor strength		
	r	*p*-value	β	r	*p*-value	β
HV angle	1.000			−0.315	0.002**	0.121
Toe flexor strength	−0.315	0.002**	0.121	1.000		
FR	−0.007	0.949	0.950	0.198	0.056	0.515
MSL	−0.129	0.217	0.764	0.330	0.001**	0.092
Plantar pressure						
Gait						
T1	0.253	0.014*	0.301	−0.038	0.718	0.935
T25	−0.362	< 0.001**	0.047	0.226	0.029*	0.403
M1	−0.138	0.186	0.736	0.013	0.899	0.948
M24	−0.242	0.019*	0.342	−0.067	0.520	0.901
M5	−0.091	0.385	0.860	−0.007	0.944	0.949
MF	0.163	0.116	0.650	−0.211	0.041*	0.463
MH	−0.168	0.106	0.632	−0.065	0.532	0.904
LH	0.301	0.003**	0.154	−0.132	0.205	0.754
FR						
T1	0.028	0.791	0.942	0.223	0.031*	0.415
T25	−0.130	0.213	0.762	0.199	0.054	0.509
M1	0.308	0.002**	0.137	−0.135	0.195	0.745
M24	0.124	0.235	0.779	−0.318	0.002**	0.115
M5	−0.144	0.166	0.716	−0.095	0.364	0.852
MF	0.212	0.040*	0.459	−0.348	0.001**	0.064
MH	−0.174	0.093	0.608	−0.193	0.062	0.534
LH	−0.198	0.056	0.517	−0.173	0.095	0.612

*FR* functional reach, *MSL* maximum step length, *HV* hallux valgus, *T1* first toe, *T25* second through fifth toes, *M1* first metatarsal, *M24* second through fourth metatarsals, *M5* fifth metatarsal, *MF* midfoot, *MH* medial heel, *LH* lateral heel

* *p* < 0.05, ** *p* < 0.01

## Discussion

There was no difference in FR and MSL between the HV and no HV groups. The anterior limit of the functional base of support was significantly correlated with toe flexor strength [20]. On the other hand, the maximum balance test that participants leaned forward then backward as far as possible from the ankle without moving their feet is heavily involved with ankle inversion-eversion ROM, and the maximal balance range was not influenced by the presence or absence of HV [11]. As for the reason why there is no difference between FR and MSL in HV and no HV groups, young women with HV may be adequately compensated using different balance strategies, despite toe flexor strength with HV weakness.

There was a difference in toe flexor strength between the HV and no HV groups. Also, HV angle and toe flexor strength were negatively correlated. It has often been reported that plantar flexion strength of HV decreases in older people [5–7]. The valgus / pronation of the first phalanx associated with HV may relatively shorten the length of the flexor hallucis muscles, making it difficult to exert the toe flexor strength. Although it was not possible to evaluate the muscle strengths of the hallux and lesser toes separately with the equipment used, it became clear that even in young people, plantar flexion strength was lower in those with HV.

There was no significant difference in pressure on the first toe during walking between the HV and no HV groups. There are various reports on the pressure of the toes during walking in older people. The HV showed no significant difference in plantar pressure of hallux during walking and the pressures of the first and second metatarsals were significantly higher than those of no HV [13]. Many reports suggest that the first toe pressure on HV during walking is low [7, 8, 28, 29]. Greater HV severity was associated with great toe pain and reduced loading under the hallux when walking [29]. Conversely, the pressure of the hallux during walking was significantly higher in women with mild HV than in those with no history of foot or lower limb problems [30]. The participants of this study were young persons with no pain in the toes and no disability in the lower limbs. That is, the participants did not avoid pain and were not affected by other joint disorders. Even the HV may not have affected the pressure of the hallux during walking.

Furthermore, in the HV group, the pressures of the second through fifth toes and the second through fourth metatarsals during walking were significantly lower, and the pressure of the lateral heel was significantly higher than that in no HV. From this result, it is considered that the load is applied to the outside by heel contact, and the load is decreased from mid stance to toe off. In HV, the ratio of the center of pressure excursion to the foot width during walking is low, and it is pointed out that foot pronation may occur [28]. In this study, although the center of pressure and foot posture were not measured, the tendency toward foot pronation in the hallux valgus is predicted due to the decrease in lateral pressure during walking.

In FR, when body weight was moved forward while standing on both legs, the pressure on the first metatarsal in HV was higher compared to no HV group, but not the pressure on the hallux and lesser toes. The varus of the first metatarsal bone and the valgus / pronation of the first phalanx that is associated with HV may increase the most prominent pressure on the first metatarsophalangeal joint.

Toe flexor strength and MSL were positively correlated. However, toe flexor strength and FR were not correlated. Long and short toe flexor muscles produce maximal voluntary moments around the metatarsal phalangeal joints in 0°–10° ankle dorsiflexion and in 25°–45° metatarsal phalangeal joint dorsiflexion [31]. Flexor muscles exert their most tension when the muscle length becomes long due to the two joint movements. The movement of MST includes metatarsal phalangeal joint dorsiflexion, but the movement of FR does not include this joint movement. Also, similar to FR, previous studies have reported that maximal balance range does not depend on the presence or absence of HV [11], with the most important independent predictor noted as ankle dorsiflexion strength [10] and ankle inversion-eversion ROM [11]. Therefore, in MST, more toe flexor muscles were acting, but in FR, it may not have been acting. That is, it is considered that there was a significant relationship only between toe flexor strength and MSL.

This study has some limitations. First, an HV angle of 16° or more indicated HV, as determined by the footprint. This angle would correspond to 20° on X-ray. Therefore, an X-ray image showing an angle of 15° may indicate mild HV, and it is possible that the no HV group included participants with mild HV. Second, pressure of the supporting foot in MSL could not be measured by the foot analyzer used in this study, and the pressure distribution with or without HV remains unknown. Third, FR and MSL were measured as functional activities. These movements involve whole-body movements, and although their relationship with toe function was recognized, involvement of other parts of the body other than the toes could not be ruled out. A relationship was observed between HV angle and toe flexor strength, and between toe flexor strength and MSL, but the causal relationship remains unknown.

## Conclusions

In conclusion, the main finding of this study was that in young females with HV, toe flexor strength is significantly reduced and positively correlated with MSL. HV alters foot loading patterns during walking and pressure distribution during FR. Foot problems, including hallux valgus, are associated with falls in old age. Attention should be paid to the onset and deterioration of hallux valgus from an early age.

## Data Availability

Not applicable.

## Abbreviations

HV: Hallux valgus
FR: Functional reach
MSL: Maximum step length
