# Correction to: Correlation between functional ability, toe flexor strength, and plantar pressure of hallux valgus in young female adults: a cross-sectional study

**DOI:** 10.1186/s13047-020-00419-7

**Published:** 2020-08-07

**Authors:** Mieko Yokozuka, Kanako Okazaki, Yuko Sakamoto, Koko Takahashi

**Affiliations:** 1 Preparing Section for New Faculty of Medical Science, Fukushima Medical, University, 1 Hikariga-oka, Fukushima, 960-1295 Japan; 2 School of Nursing, Fukushima Medical University, 1 Hikariga-oka, Fukushima, 960-1295 Japan

**Correction to: J Foot Ankle Res (2020) 13: 44**

**https://doi.org/10.1186/s13047-020-00411-1**

Following publication of the original article [1] the authors have notified us of an error in the first row of Table 1:

Incorrect values: 20.3 ± 5.1(4–15) 11.0 ± 2.6(16–43)

Correct values: 20.3 ± 5.1(16–43) 11.0 ± 2.6(4–15)
